# Elecsys Cerebrospinal Fluid Immunoassays Accurately Detect Alzheimer’s Disease Regardless of Concomitant Small Vessel Disease

**DOI:** 10.3233/JAD-221187

**Published:** 2023-06-13

**Authors:** Marion Ortner, Korbinian Lanz, Oliver Goldhardt, Felix Müller-Sarnowski, Janine Diehl-Schmid, Hans Förstl, Dennis M. Hedderich, Igor Yakushev, Chad A. Logan, Jan-Philipp Weinberger, Maryline Simon, Timo Grimmer

**Affiliations:** a Department of Psychiatry and Psychotherapy, Klinikum rechts der Isar, Technical University of Munich, School of Medicine, Munich, Germany; b Department of Neuroradiology, Klinikum rechts der Isar, Technical University of Munich, School of Medicine, Munich, Germany; c Department of Nuclear Medicine, Klinikum rechts der Isar, Technical University of Munich, School of Medicine, Munich, Germany; d Roche Diagnostics GmbH, Penzberg, Germany; e Roche Diagnostics International Ltd, Rotkreuz, Switzerland

**Keywords:** Alzheimer’s disease, biomarkers, cerebral small vessel diseases, cerebrospinal fluid, diagnosis, differential

## Abstract

**Background:**

Differentiating dementia due to small vessel disease (SVD) from dementia due to Alzheimer’s disease (AD) with concomitant SVD is challenging in clinical practice. Accurate and early diagnosis of AD is critical to delivering stratified patient care.

**Objective:**

We characterized the results of Elecsys^®^ cerebrospinal fluid (CSF) immunoassays (Roche Diagnostics International Ltd) in patients with early AD, diagnosed using core clinical criteria, with varying extent of SVD.

**Methods:**

Frozen CSF samples (*n* = 84) were measured using Elecsys β-Amyloid(1–42) (Aβ_42_), Phospho-Tau (181P) (pTau181), and Total-Tau (tTau) CSF immunoassays, adapted for use on the cobas^®^ e 411 analyzer (Roche Diagnostics International Ltd), and a robust prototype β-Amyloid(1–40) (Aβ_40_) CSF immunoassay. SVD was assessed by extent of white matter hyperintensities (WMH) using the lesion segmentation tool. Interrelations between WMH, biomarkers, fluorodeoxyglucose F18-positron emission tomography (FDG-PET), and other parameters (including age and Mini-Mental State examinations [MMSE]) were assessed using Spearman’s correlation, sensitivity/specificity, and logistic/linear regression analyses.

**Results:**

The extent of WMH showed significant correlation with Aβ_42_/Aβ_40_ ratio (Rho=-0.250; *p* = 0.040), tTau (Rho = 0.292; *p* = 0.016), tTau/Aβ_42_ ratio (Rho = 0.247; *p* = 0.042), age (Rho = 0.373; *p* = 0.002), and MMSE (Rho=-0.410; *p* = 0.001). Sensitivity/specificity point estimates for Elecsys CSF immunoassays versus FDG-PET positivity for underlying AD pathophysiology were mostly comparable or greater in patients with high versus low WMH. WMH were not a significant predictor and did not interact with CSF biomarker positivity but modified the association between pTau181 and tTau.

**Conclusion:**

Elecsys CSF immunoassays detect AD pathophysiology regardless of concomitant SVD and may help to identify patients with early dementia with underlying AD pathophysiology.

## INTRODUCTION

Alzheimer’s disease (AD), the most common cause of dementia, is a progressive neurodegenerative disease that is typically recognized by initial memory impairment and cognitive decline, followed by the deterioration of language and behavioral functions, visuospatial orientation, and the motor system [1]. AD is defined by neuropathological changes, namely neuritic plaques containing amyloid-β (Aβ) peptides and neurofibrillary tangles containing aggregated tau proteins, which ultimately lead to neuronal injury and degeneration [2]. While the underlying neuropathology of AD is well defined, the clinical presentation of the disease is heterogeneous; approximately 25% of AD cases do not conform to typical AD presentation [3]. Multiple stages of AD have been identified including pre-symptomatic stage AD, patients with subjective cognitive decline who exhibit AD biomarkers, mild cognitive impairment (MCI) due to AD, mild dementia due to AD, and moderate-to-severe AD [3–6]. MCI due to AD and mild dementia due to AD are often classed as “early AD” [7, 8].

The core AD biomarkers can be classified into two groups: biomarkers of Aβ peptide deposition, including decreased levels of Aβ_42_ in cerebrospinal fluid (CSF) and amyloid positivity using positron emission tomography (PET), and biomarkers of neuronal injury and degeneration, including elevated levels of phospho-Tau181 (pTau181) and total Tau (tTau) in CSF, decreased fluorodeoxyglucose F18 (FDG) uptake on PET, and disproportionate atrophy on structural magnetic resonance imaging [9]. Numerous clinical studies have demonstrated that CSF biomarkers are associated with AD pathology and have demonstrated the ability to accurately identify AD at the stage of incipient dementia [10–12]. Thus, CSF biomarkers have been incorporated into multiple diagnostic frameworks for AD [13–15].

The accurate and early diagnosis of AD is critical to delivering stratified patient care and is a key consideration for current clinical trials evaluating novel treatments targeting AD neuropathology [16]. Many existing CSF immunoassays for AD are limited by lot-to-lot and interlaboratory variations, which have hindered the widespread introduction of CSF biomarkers into clinical practice [17]. The fully automated Elecsys^®^ CSF immunoassays (Roche Diagnostics International Ltd, Rotkreuz, Switzerland) accurately detect amyloid positivity by determining CSF pTau181/Aβ_42_ and tTau/Aβ_42_ biomarker ratios and have demonstrated superior interlaboratory variation (coefficient of variation: 4%) compared with existing manual CSF assays (coefficient of variation: >15%) [18]. The Elecsys CSF immunoassays can also accurately predict future disease progression and, thus, have the potential to support the diagnosis of early AD [18].

Though dementia due to AD is the most common form of dementia, less than half of cases are expected to be solely caused by AD and most cases are expected to be mixed dementias [19]. AD frequently coexists with other neurodegenerative co-pathologies, for example, vascular disease including cerebral small vessel disease (SVD) and large vessel disease [2, 20]. Vascular disease is thought to play a major role in the pathogenesis of AD [20]. SVD affects small arteries, arterioles, veins, and capillaries of the brain, and is the most frequent cause of vascular dementia [21]. The disease is characterized by white matter hyperintensities (WMH), small subcortical infarcts, lacunes, enlarged perivascular spaces, microbleeds, and brain atrophy [22]. WMH volume, particularly in parietal regions, is elevated among individuals with and at risk of AD [23]. Additionally, the presence of WMH increases the risk of cognitive decline and AD and has been shown to contribute to disease progression and severity [24–26]. Differentiating dementia due to SVD from dementia due to AD with concomitant SVD is challenging in clinical practice [27]. CSF biomarker levels could be altered due to impaired cerebral drainage caused by SVD [28]. In patients with early dementia, it is important to differentiate those with or without underlying AD, as the correct diagnosis is critical to delivering stratified patient care, particularly as novel disease-modifying treatments (DMTs) for AD (e.g., anti-Aβ drugs) are thought to be most effective in the early stages of the disease [29]. For example, a patient diagnosed with early dementia due to underlying AD may be optimally treated with anti-Aβ DMTs, whereas a patient with early dementia due to SVD may be optimally treated with a potential intervention for SVD such as endothelin antagonists, neurotrophins, or phosphodiesterase inhibitors [30].

This study aimed to characterize the results of Elecsys CSF immunoassays in patients diagnosed with early AD (based on core AD clinical criteria), with or without FDG-PET positivity for underlying AD pathophysiology, and with varying extent of SVD, and to identify a possible relationship between WMH and parameters of Elecsys CSF immunoassays.

## METHODS

### Study design

This study was conducted at a single center in Munich, Germany (the Outpatient Clinic at the Centre for Cognitive Disorders, Department of Psychiatry, Klinikum rechts der Isar, Technical University of Munich, School of Medicine) between July 2019 and July 2020. Patients with early AD, i.e., MCI or mild dementia due to AD based on core AD clinical criteria, were enrolled in the study. The patient population comprised the target population of a number of clinical trials in patients with early AD [31–35]. Patient samples were retrospectively collected from the study center biobank.

### Ethics approval and consent to participate

The study was submitted to and approved by the Ethics Committee of the Technical University of Munich, Munich, Germany (Project Code: 312/19 S). All participants provided written consent for the research use of their data and the study was performed according to the principles of the Declaration of Helsinki.

### Diagnosis of AD and SVD

Patients with early AD were diagnosed based on expert opinion using core AD clinical criteria (patients did not have substantial concomitant cerebrovascular disease, defined by a history of a stroke temporally related to the onset or worsening of cognitive impairment; or the presence of multiple or extensive infarcts or severe WMH burden) and the global Clinical Dementia Rating (CDR) scale [9, 36]. The severity of cognitive impairment was determined by global CDR and CDR sum of boxes scores [36]. Patients who scored 0.5 on the global CDR were diagnosed with MCI due to AD and patients who scored 1.0 were diagnosed with mild dementia due to AD. Patients with early AD were evaluated using neuro-psychological evaluation including Mini-Mental State examinations (MMSE) and the Consortium to Establish a Registry for Alzheimer’s Disease Neuropsychological Assessment Battery [37, 38]. Routine laboratory screening tests were performed for further examination of cognitive impairment, including CSF analyses. Underlying AD pathophysiology was identified by a typical metabolic pattern using FDG-PET, which was considered as the ‘standard-of-truth’ in this study, and SVD was fully automatically assessed by the extent of WMH on a three-dimensional fluid-attenuated inversion recovery magnetic resonance imaging scan using the lesion segmentation tool [39].

### CSF samples and biomarker measurement

For each patient, 5–8 ml of CSF were acquired by lumbar puncture, between the L3/L4 or L4/L5 intervertebral space, using atraumatic cannulas and collected in two sterile polypropylene tubes. Immediately after collection, CSF from one polypropylene tube was centrifuged at 2000 x *g* for 10 min at 4°C. Aliquots were frozen and stored at –80°C at the study center biobank, prior to measurement with the Elecsys CSF immunoassays. The site-specific pre-analytical protocol did not fully adhere to the Elecsys CSF immunoassay method sheets as the type of polypropylene tubes used and size of the aliquots (0.5 ml) varied. CSF from the second polypropylene tube was used to determine routine parameters including cell count, glucose and lactate measurement, total protein content, and CSF/serum ratio of albumin.

Patient CSF samples were measured using modified versions of the respective Conformité Européen approved Elecsys β-Amyloid(1–42) CSF, Phospho-Tau (181P) CSF, and Total-Tau CSF immunoassays, adapted for use on the cobas^®^ e 411 analyzer, and a robust prototype β-Amyloid(1–40) CSF immunoassay that is for investigational use only and not commercially available (Roche Diagnostics International Ltd). These assays are fully automated electrochemiluminescence immunoassays, which utilize monoclonal antibodies in the form of a sandwich test principle. Patient CSF samples were tested for amyloid positivity by calculating pTau181/Aβ_42_ and tTau/Aβ_42_ biomarker ratios from the corresponding immunoassay measurements for Aβ_42_, pTau181, and tTau. For post-hoc analyses, an Aβ_42_/_40_ ratio was calculated from measurements for Aβ_42_ and Aβ_40_.

### Statistical analyses

All data were analyzed using the statistical platform software IBM SPSS Statistics Version 26. Missing data were not imputed.

Spearman correlation analyses were employed to determine the non-parametric correlation between the extent of WMH and Elecsys CSF immunoassay biomarker results (Aβ_42_, Aβ_42_/Aβ_40_ ratio, pTau181, tTau, pTau181/Aβ_42_ ratio, and tTau/Aβ_42_ ratio), age, MMSE, and clinical severity measured using CDR sum of boxes. A *p*-value of <0.05 indicated statistical significance.

The ‘sensitivity’ and ‘specificity’ of the Elecsys CSF immunoassays—in this case, the ‘positive percent agreement’ and ‘negative percent agreement’, respectively, of the immunoassays (as FDG-PET is not an accepted reference standard for detecting AD, but was the ‘standard-of-truth’ in the present analysis)—were calculated for the whole study group and for patients stratified into tertiles according to volume of WMH, denoted as low (<0.5 ml), medium (0.5–2.5 ml), and high (>2.5 ml). The cut-off values of 0.5 ml and 2.5 ml were chosen to generate subgroups with similar sample sizes and, in accordance with the core AD clinical criteria, no patients presented with severe WMH burden. Post-hoc analyses were carried out for sensitivity and specificity using CSF Aβ_42_/Aβ_40_ positivity as the standard-of-truth. Aβ_42_/Aβ_40_ positivity presumably develops earlier during AD compared with FDG-PET positivity; the cut-off (<0.048) for Aβ_42_/Aβ_40_ positivity was established in comparison with amyloid PET positivity in an independent cohort [40, 41].

Multivariate logistic regression analyses were employed to assess the effect of WMH on the interrelations between Elecsys CSF immunoassay biomarker positivity and FDG-PET positivity. Multivariate linear regression analyses were also employed to investigate the effect of WMH on the interrelations between Elecsys CSF immunoassay biomarkers for the whole study group and for a subgroup of patients showing amyloid positivity (defined by CSF Aβ_42_/Aβ_40_ positivity). The regression models were selected based on the temporal sequence of biomarker positivity during the course of AD; pTau181 and tTau were used as the dependent variables, while biomarkers and biomarker ratios that are further upstream in the amyloid cascade were used as the independent variables, in addition to WMH and the corresponding interaction term (i.e., biomarker x WMH). A priori factors—age, sex, MMSE, and clinical severity measured using CDR sum of boxes—were assessed as potential covariates or confounders based on an increase in adjusted R^2^. Two-parameter interactions were assessed to increase the model fit. Independent associations for these factors were assessed in univariate linear models. For both the multivariate logistic regression analyses and the multivariate and univariate linear regression analyses, a *p*-value of <0.05 indicated statistical significance.

## RESULTS

### Patient characteristics

In total, 84 patients who met the core clinical criteria for early AD were enrolled in the study (male *n* = 37 [44% ]; mean age [standard deviation] = 64.6 [9.87] years). Patient characteristic data for the whole group, stratified by WMH (low [*n* = 30], medium [*n* = 27], and high [*n* = 27]), are shown in Table 1. Of the 84 patients with early AD, 26 patients (30.95%) were diagnosed with MCI (CDR = 0.5) and 58 patients (69.05%) were diagnosed with mild dementia due to AD (CDR = 1.0).

**Table 1:** Patient characteristics for the whole group and stratified by WMH

Characteristic	Whole group^a^ (*n* = 84)	WMH low (<0.5 ml; *n* = 30)	WMH medium (0.5–2.5 ml; *n* = 27)	WMH high (>2.5 ml; *n* = 27)
Mean age, y (SD)	64.6 (9.87)	58.1 (9.30)	66.6 (7.71)	69.7 (8.71)
Median age, y (IQR)	64.5 (56.25–74)	56 (50–64.25)	64 (61–74)	72 (67–76)
Male, *n* (%)	37 (44.0)	17 (56.7)	11 (40.7)	9 (33.3)
Mean CDR global (SD)	0.71 (0.31)	0.63 (0.22)	0.76 (0.35)	0.74 (0.35)
Median CDR global (IQR)	0.5 (0.5–1)	0.5 (0.5–1)	0.5 (0.5–1)	0.5 (0.5–1)
Mean CDR SOB (SD)	3.65 (2.43)	3.06 (1.32)	3.85 (2.63)	4.15 (3.13)
Median CDR SOB (IQR)	3 (2.125–4.875)	2.5 (2.5–3.5)	3.5 (1.5–5.0)	3.5 (2.5–4.5)
Mean WMH, ml (SD)	4.03 (8.60)	0.20 (0.15)	1.21 (0.57)	11.11 (12.59)
Median WMH, ml (IQR)	0.952 (0.268–3.192)	0.192 (0.074–0.346)	1.072 (0.824–1.448)	5.080 (3.208–17.832)
Mean Aβ_42_, pg/ml (SD)	857.54 (404.96)	955.53 (463.58)	798.19 (371.25)	808.02 (358.75)
Median Aβ_42_, pg/ml (IQR)	715.6 (553.15–1,074.5)	752.6 (584.475–1,509.5)	687.5 (517.4–921.5)	750.4 (518.0–1,041.0)
Mean Aβ_40_, pg/ml (SD)	16,301.38	15,712.70	16,940.78	16,316.07
	(4,797.51)	(4,944.30)	(4,986.00)	(4,530.23)
Median Aβ_40_, pg/ml (IQR)	16,354.5	14,861.5	17,306	15,359
	(12,977.25–19,158.5)	(12,196.5–18,521)	(13,571–20,199)	(13,738–18,342)
Mean pTau181, pg/ml (SD)	32.67 (18.94)	28.08 (16.20)	37.10 (19.19)	33.34 (20.95)
Median pTau181, pg/ml (IQR)	30.09 (19.39–37.785)	26.485 (15.65–33.9125)	32.85 (22.26–44.49)	30.65 (19.30–39.78)
Mean tTau, pg/ml (SD)	334.82 (177.68)	286.46 (142.07)	370.20 (168.51)	353.18 (213.38)
Median tTau, pg/ml (IQR)	313.05	278.00	329.60	337.10
	(226.50–367.75)	(172.225–329.75)	(238.70–469.80)	(235.30–398.40)
Mean pTau181/Aβ_42_ (SD)	0.05 (0.04)	0.04 (0.03)	0.05 (0.03)	0.05 (0.05)
Median pTau181/Aβ_42_ (IQR)	0.04 (0.02–0.06)	0.04 (0.01–0.05)	0.05 (0.03–0.07)	0.03 (0.03–0.07)
Mean tTau/Aβ_42_ (SD)	0.49 (0.37)	0.39 (0.29)	0.52 (0.25)	0.57 (0.52)
Median tTau/Aβ_42_ (IQR)	0.39 (0.27–0.63)	0.38 (0.13–0.48)	0.52 (0.25–0.65)	0.35 (0.30–0.68)
Mean Aβ_42_/Aβ_40_ (SD)	0.05 (0.02)	0.06 (0.02)	0.05 (0.02)	0.05 (0.02)
Median Aβ_42_/Aβ_40_ (IQR)	0.05 (0.04–0.06)	0.05 (0.04–0.09)	0.04 (0.04–0.05)	0.04 (0.04–0.05)
FDG-PET subgroup^b^, *N*	72	28	24	20
FDG-PET positivity for AD, *n* (%)	42 (58.3)	14 (50.0)	15 (62.5)	13 (65.0)

Aβ, amyloid-beta; AD, Alzheimer’s disease; CDR, Clinical Dementia Rating scale; CSF, cerebrospinal fluid; FDG-PET, fluorodeoxyglucose F18-positron emission tomography; IQR, interquartile range; MCI, mild cognitive impairment; *n*, number; pTau181, phospho-Tau181; SD, standard deviation; SOB, sum of boxes scores; tTau, total Tau; WMH, white matter hyperintensities; y, years. ^a^Of the 84 patients with early AD enrolled in the study, 26 patients were diagnosed with MCI (CDR = 0.5) and 58 patients were diagnosed with mild dementia due to AD (CDR = 1.0). ^b^Patients with available FDG-PET results.

FDG-PET results were available for 72 patients. Patient characteristic data for the FDG-PET subgroup, stratified by WMH (low [*n* = 28], medium [*n* = 24], and high [*n* = 20]), are shown in Supplementary Table 1. Of the 72 patients in the FDG-PET subgroup, 42 (58.3%) patients showed FDG-PET positivity indicative of underlying AD pathophysiology.

### Correlations between WMH and Elecsys CSF immunoassay parameters

Spearman’s correlation analyses showed that the extent of WMH were significantly associated with the following parameters: Aβ_42_/Aβ_40_ ratio (Rho=-0.250; *p* = 0.040), tTau (Rho = 0.292; *p* = 0.016), tTau/Aβ_42_ ratio (Rho = 0.247; *p* = 0.042), age (Rho = 0.373; *p* = 0.002), and MMSE (Rho=-0.410; *p* = 0.001) (Table 2).

**Table 2:** Spearman’s correlation analyses between WMH and parameters of Elecsys CSF immunoassays

Parameter	Spearman’s correlation	
	Rho	*p*
Aβ_42_	–0.190	0.120
Aβ_42_/Aβ_40_ ratio	–0.250	0.040*
pTau181	0.216	0.076
tTau	0.292	0.016*
pTau181/Aβ_42_ ratio	0.231	0.058
tTau/Aβ_42_ ratio	0.247	0.042*
Age	0.373	0.002*
MMSE	–0.410	0.001*
CDR SOB	0.225	0.065

Aβ, amyloid-beta; CDR SOB, Clinical Dementia Rating scale sum of boxes scores; CSF, cerebrospinal fluid; MMSE, Mini-Mental State examinations; pTau181, phospho-Tau181; tTau, total Tau; WMH, white matter hyperintensities. *Indicates statistical significance (*p* <0.05).

### Sensitivity and specificity of Elecsys CSF immunoassays in relation to extent of WMH

Most point estimates for sensitivity and specificity of Elecsys CSF immunoassays compared with FDG-PET positivity as standard-of-truth, were comparable or greater in patients with high WMH (*n* = 20) compared with patients with low WMH (*n* = 28); however, the opposite trend was observed for point estimates for specificity of pTau181/Aβ_42_ ratio and tTau/Aβ_42_ ratio (Table 3). Across all WMH subgroups, the point estimates for specificity were relatively low compared to the point estimates for sensitivity; however, both sensitivity and specificity point values predominantly remained within 95% confidence intervals for the group point estimate.

**Table 3:** Sensitivity and specificity of Elecsys CSF immunoassays stratified by WMH compared with FDG-PET positivity

Biomarker	Sensitivity, %				Specificity, %			
	WMH	WMH,	WMH	FDG-PET	WMH	WMH	WMH	FDG-PET
	low,	medium,	high,	subgroup^a^	low,	medium,	high,	subgroup^a^
	*n* = 28	*n* = 24	*n* = 20	(95% CI), *n* = 72	*n* = 28	*n* = 24	*n* = 20	(95% CI), *n* = 72
Aβ_42_	85.7	93.3	84.6	88.1 (73.6–95.5)	53.8	44.4	57.1	50.0 (31.7–68.3)
pTau181	64.3	60.0	76.9	66.7 (50.4–80.0)	71.4	55.6	71.4	66.7 (47.1–82.1)
tTau	64.3	60.0	84.6	69.0 (52.8–81.9)	71.4	55.6	71.4	66.7 (47.1–82.1)
pTau181/Aβ_42_ ratio	85.7	100.0	92.3	92.9 (79.4–98.1)	64.3	44.4	57.1	56.7 (37.7–74.0)
tTau/Aβ_42_ ratio	85.7	93.3	92.3	90.5 (76.5–97.0)	64.3	44.4	57.1	56.7 (37.7–74.0)

Aβ, amyloid-beta; CI, confidence interval; CSF, cerebrospinal fluid; FDG-PET, fluorodeoxyglucose F18-positron emission tomography; *n*, number; pTau181, phospho-Tau181; tTau, total Tau; WMH, white matter hyperintensities. ^a^Patients with available FDG-PET results.

In post-hoc analyses, the point estimates for sensitivity and specificity of Elecsys CSF immunoassays compared with CSF Aβ_42_/Aβ_40_ positivity as standard-of-truth (Table 4), were mostly greater than those compared with FDG-PET positivity as standard-of-truth (Table 3). Across all WMH subgroups, the point estimates for both sensitivity and specificity point values predominantly remained within 95% confidence intervals for the group point estimate. However, for Aβ_42_, the point estimate for specificity in patients with high WMH compared with FDG-PET positivity (57.1%) was higher than when compared with CSF Aβ_42_/Aβ_40_ positivity (44.4%).

**Table 4:** Sensitivity and specificity of Elecsys CSF immunoassays stratified by WMH compared with CSF Aβ42/Aβ40 positivity^a,b^

Biomarker	Sensitivity, %				Specificity, %			
	WMH	WMH	WMH	FDG-PET	WMH	WMH	WMH	FDG-PET
	low,	medium,	high,	subgroup^c^	low,	medium,	high,	subgroup^c^
	*n* = 28	*n* = 24	*n* = 20	(95% CI), *n* = 72	*n* = 28	*n* = 24	*n* = 20	(95% CI), *n* = 72
Aβ_42_	100.0	93.8	81.8	92.5 (78.5–98.0)	60.0	50.0	44.4	53.1 (35.0–70.5)
pTau181	69.2	75.0	90.9	77.5 (61.1–88.6)	73.3	87.5	77.8	78.1 (59.6–90.1)
tTau	69.2	81.3	90.9	80.0 (63.9–90.4)	73.3	100.0	66.7	78.1 (59.6–90.1)
pTau181/Aβ_42_ ratio	100.0	100.0	100.0	100.0 (89.1–100.0)	73.3	50.0	55.6	62.5 (43.7–78.3)
tTau/Aβ_42_ ratio	100.0	100.0	100.0	100.0 (89.1–100.0)	73.3	62.5	55.6	65.6 (46.8–80.9)

Aβ, amyloid-beta; CI, confidence interval; CSF, cerebrospinal fluid; FDG-PET, fluorodeoxyglucose F18-positron emission tomography; *n*, number; pTau181, phospho-Tau181; tTau, total Tau; WMH, white matter hyperintensities. ^a^Amyloid positivity based on Aβ_42_/Aβ_40_ ratio <0.048, which optimally differentiated amyloid PET positivity in an independent cohort [40, 41]. ^b^Post-hoc analyses. ^c^Patients with available FDG-PET results.

### Effect of WMH on the interrelations between Elecsys CSF immunoassay biomarker positivity and FDG-PET positivity

The multivariate logistic regression analyses for the association between Elecsys CSF immunoassay biomarker positivity with FDG-PET positivity showed that WMH were not a significant predictor and did not interact with biomarker positivity (Table 5).

**Table 5:** Multivariate logistic regression analyses between FDG-PET positivity, Elecsys CSF immunoassay biomarker positivity, and WMH

Dependent variable	Nagelkerkes R^2^	Independent variables	FDG-PET subgroup (*n* = 72)^a^	
			beta	*p*
FDG-PET positivity	0.224	Aβ_42_ positivity	6.869	0.003*
		WMH	0.969	0.677
		Aβ_42_ positivity x WMH	1.025	0.775
FDG-PET positivity	0.159	pTau181 positivity	3.258	0.036*
		WMH	0.953	0.463
		pTau181 positivity x WMH	1.073	0.442
FDG-PET positivity	0.180	tTau positivity	3.600	0.024*
		WMH	0.948	0.470
		tTau positivity x WMH	1.080	0.437
FDG-PET positivity	0.325	tTau/Aβ_42_ positivity	12.184	<0.001*
		WMH	0.962	0.717
		tTau/Aβ_42_ positivity x WMH	1.019	0.869
FDG-PET positivity	0.365	pTau181/Aβ_42_ positivity	16.828	<0.001*
		WMH	9.650	0.753
		pTau181/Aβ_42_ positivity x WMH	1.015	0.904

Aβ, amyloid-beta; CSF, cerebrospinal fluid; FDG-PET, fluorodeoxyglucose F18-positron emission tomography; n, number; pTau181, phospho-Tau181; tTau, total Tau; WMH, white matter hyperintensities. ^a^Patients with available FDG-PET results. *Indicates statistical significance (*p* < 0.05).

### Effect of WMH on the interrelations between Elecsys CSF immunoassay biomarkers

The multivariate linear regression analyses for the association between WMH and Elecsys CSF immunoassay biomarker results showed WMH were a significant predictor for, and modified, the association of pTau181 on tTau (Table 6): WMH (beta = 1.220; *p* = 0.001) and pTau181 x WMH (beta = 0.216; *p* = 0.001) (whole study group); WMH (beta = 0.532; *p* = 0.292) and pTau181 x WMH (beta = 0.199, *p* = 0.016) (amyloid positive subgroup) (Supplementary Table 2). However, univariate linear regression analyses showed WMH were not an independent predictor for tTau alone (beta = 0.229; *p* = 0.060) (Supplementary Table 3).

**Table 6:** Multivariate linear regression analyses between Elecsys CSF immunoassay biomarkers and WMH

Dependent variable	corrR^2^	Independent variables	Whole group (*n* = 84)	
			beta	*p*
pTau181	0.102	Aβ_42_	–0.352	0.008*
		WMH	–0.032	0.905
		Aβ_42_ x WMH	0.163	0.529
pTau181	0.116	Aβ_40_	0.299	0.029*
		WMH	0.085	0.792
		Aβ_40_ x WMH	0.174	0.586
pTau181	0.471	Aβ_42_/Aβ_40_	–2.144	0.001*
		WMH	0.294	0.875
		Aβ_42_/Aβ_40_ x WMH	–0.050	0.941
		Age	–0.775	0.008*
		Age x WMH	–0.663	0.656
		CDR SOB	–0.144	0.273
		CDR SOB x WMH	0.695	0.042*
		Aβ_42_/Aβ_40_ x Age	1.729	0.014*
pTau181	0.776	tTau/Aβ_42_	1.057	<0.001*
		WMH	0.051	0.703
		tTau/Aβ_42_ x WMH	–0.371	0.058
tTau	0.069	Aβ_42_	–0.269	0.046*
		WMH	0.004	0.987
		Aβ_42_ x WMH	0.177	0.501
tTau	0.144	Aβ_40_	0.324	0.017*
		WMH	0.125	0.692
		Aβ_40_ x WMH	0.163	0.603
tTau	0.281	Aβ_42_/Aβ_40_	–0.381	0.003*
		WMH	1.064	0.056
		Aβ_42_/Aβ_40_ x WMH	–0.950	0.081
tTau	0.971	pTau181	0.898	<0.001*
		WMH	1.220	0.001*
		pTau181 x WMH	0.216	0.001^*^
		Age	0.091	<0.001*
		Age x WMH	–1.277	<0.001*
		CDR SOB	0.043	0.119
		CDR SOB x WMH	–0.134	0.062
tTau	0.729	pTau181/Aβ_42_	0.886	<0.001*
		WMH	–0.118	0.405
		pTau181/Aβ_42_ x WMH	0.022	0.898

Aβ, amyloid-beta; CDR SOB, Clinical Dementia Rating scale sum of boxes scores; CSF, cerebrospinal fluid; *n*, number; pTau181, phospho-Tau181; tTau, total Tau; WMH, white matter hyperintensities. *Indicates statistical significance (*p* < 0.05).

## DISCUSSION

In patients with early AD, it is important to differentiate those with underlying AD pathophysiology regardless of the extent of SVD as the correct diagnosis is critical to delivering stratified patient care. We aimed to characterize the results of Elecsys CSF immunoassays for detecting underlying AD pathophysiology in patients with early AD (based on core AD clinical criteria) with varying extent of concomitant SVD (assessed by the volume of WMH). We also aimed to identify a possible relationship between WMH and parameters of Elecsys CSF immunoassays. In patients with early AD, the extent of WMH showed significant correlation with Aβ_42_/Aβ_40_ ratio, tTau, and tTau/Aβ_42_ ratio, suggesting a possible confounding effect on the performance of Elecsys CSF immunoassays. The point estimates for sensitivity and specificity of the CSF immunoassays, compared with FDG-PET positivity as standard-of-truth, were generally comparable or greater in patients with high WMH versus patients with low WMH, suggesting that the extent of WMH is unlikely to affect the performance of Elecsys CSF immunoassays in detecting AD pathophysiology. For the association between Elecsys CSF immunoassay biomarker positivity with FDG-PET positivity, WMH were not a significant predictor and did not interact with biomarker positivity, which further highlights the robustness of the immunoassays to detect AD pathophysiology, regardless of extent of WMH. However, WMH did modify the association between pTau181 and tTau. In summary, our results demonstrate that the performance of the Elecsys CSF immunoassays in detecting AD is unlikely to be affected by the presence of concomitant SVD; therefore, they have potential to help differentiate between dementia due to SVD and AD with concomitant SVD.

Point estimates for specificity compared with FDG-PET positivity were relatively low across all WMH groups in comparison to higher published specificities for CSF biomarkers compared with amyloid PET positivity. Therefore, we conducted post-hoc analyses to compare the sensitivity and specificity of the Elecsys CSF immunoassays using an alternative standard-of-truth, Aβ_42_/Aβ_40_ positivity, which presumably develops earlier during the course of AD compared with FDG-PET positivity [40]. As expected, point estimates for sensitivity and specificity compared with CSF Aβ_42_/Aβ_40_ positivity were greater than those compared with FDG-PET positivity.

This study provides further evidence that CSF biomarkers have the potential to increase the accuracy of AD diagnosis, particularly at the earlier stages of disease, as well as in cases of atypical presentation and mixed pathology, i.e., AD with concomitant SVD or large vessel disease. Though patients were diagnosed with early AD based on expert opinion at a highly specialized center using core AD clinical criteria, almost 50% did not show a typical FDG-PET pattern for AD pathophysiology, underscoring the importance of using biomarkers to support the identification of the underlying neuropathology of dementia. Ongoing drug discovery efforts focus on developing DMTs that aim to delay the onset or progression of dementia and must be initiated early in the disease process [42]. Current AD treatments provide symptomatic benefit only; as such, it is important to identify patients with AD early in the disease process, when therapies are likely to be most effective [29]. Therefore, the accurate and early identification of underlying AD pathology is vital to ensure the right diagnosis and stratified treatment, i.e., symptomatic treatments or DMTs; it is also key for patient empowerment. There is growing evidence that patients and carers wish to reach a diagnosis as soon as possible, in order to reduce the anxiety caused by symptoms and to allow patients to initiate lifestyle changes and plan for the future, e.g., implement safeguarding procedures to prevent accidents [43–47]. In addition to improving diagnostic accuracy, CSF biomarkers have the potential to facilitate health care professionals in understanding AD etiology, including AD mixed pathologies.

Since CSF biomarkers have been incorporated into various diagnostic guidelines for AD, there is a demand for accurate and reliable methods for their measurement [12–15]. Notably, in 2018, the first-generation Elecsys β-Amyloid(1–42) CSF and Phospho-Tau (181P) CSF immunoassays were granted United States Food and Drug Administration Breakthrough Device Designation to support the improved diagnosis of AD in concordance with amyloid PET visual read result [48]; this was followed by United States Food and Drug Administration approval for the second-generation Elecsys β-Amyloid(1–42) CSF II and the updated Elecsys Phospho-Tau (181P) CSF immunoassays on December 8, 2022 [49].

SVD can contribute to the pathogenesis of both vascular dementia and AD. SVD has similar pathophysiological mechanisms to AD including oxidative stress, inflammation, mitochondrial disruption, and metabolic dysfunction, as well as similar risk factors including hypertension and diabetes [27]. For these reasons, differentiating between dementia due to SVD and AD with concomitant SVD is controversial and poses a difficult challenge. Vascular dementia and AD are both leading causes of dementia; SVD potentially interacts with pathophysiological processes in AD, either independently or through additive or synergistic effects on cognitive decline [20]. Differentiating between patients with AD and patients with dementia due to SVD or vascular dementia would benefit the patient in terms of treatment; though studies have reported that the treatment and prevention for vascular dementia and SVD may benefit patients with AD, there is still lack of evidence in clinical application of treatments that benefit both AD and SVD [50]. It is possible that CSF biomarkers may share a direct relationship with SVD and AD pathology. SVD may cause alterations in CSF biomarker levels due to impaired cerebral drainage [28]. Moreover, patients with normal pressure hydrocephalus, an expansion of the CSF-filled brain ventricles, have implicated impaired function of the glymphatic system [51], which is often associated with AD pathology [52]. Although there are a number of publications linking normal pressure hydrocephalus with AD biomarkers [53, 54], further studies are warranted to validate the association between them. In this study, alterations in CSF biomarker levels due to impaired cerebral drainage may have resulted in the establishment of WMH as a significant predictor for the pTau181 and tTau biomarker association.

While the monocentric design is a key strength of this study, reducing heterogeneity and variability, this did incur a relatively small sample size (*n* = 84), though the patients were highly characterized. This study is among the first to investigate differential diagnoses in patients with early AD using CSF biomarkers; however, as there is no independent biomarker for amyloid pathology, the accurate and reliable diagnosis of early AD was a limitation. Another limitation of this study was the use of a pre-analytical protocol that was not in accordance with Elecsys CSF immunoassay method sheets; however, this was mitigated by the robustness of the biomarker and biomarker ratio cut-offs [18].

There is now increasing recognition of the importance and value to the research and clinical communities of including underserved populations in AD biomarker studies [55, 56], particularly since some of those who are disadvantaged and/or underrepresented in clinical research may have greater risks for AD neuropathology or co-pathologies such as SVD [56–59]. Thus, future studies for the purposes of validating the results of this study should include: a heterogeneous population encompassing the full spectrum of racial, ethnic, and socioeconomic backgrounds; a larger number of patients with early AD (and at earlier disease stage, i.e., asymptomatic patients at risk); and a broader study for differential diagnoses, i.e., in patients with early dementia with or without underlying AD pathophysiology and with or without concomitant large vessel disease, and in patients with SVD without AD, which may be more reflective of a real-life clinical cohort. Validation of the results in a diverse population may clarify whether different populations require different interpretations of the results, as there is evidence suggesting differences in biomarker levels between race and age, particularly in pTau181 levels [60, 61].

### Conclusion

This study demonstrated that WMH are not an effect modifier in the association between Elecsys CSF immunoassay biomarker positivity and FDG-PET positivity; thus, the performance of Elecsys CSF immunoassays in detecting AD is unlikely to be affected by the presence of concomitant SVD and may support clinicians in identifying patients with early dementia who have underlying AD pathophysiology. In patients with early dementia, accurately identifying AD is critical as novel treatments are emerging, which are likely to be most effective in early stages of the disease. Therefore, Elecsys CSF immunoassays have the potential to guide clinical decision-making for stratified patient care.

## Supplementary Material

Supplementary Material

## Data Availability

The datasets generated and/or analyzed during this study are available from the corresponding author on reasonable request. However, due to the nature of pseudonymized patient data, a material transfer agreement is required to meet ethical standards and data privacy laws of Germany.
