# Supplementary material for: Elecsys Cerebrospinal Fluid Immunoassays Accurately Detect Alzheimer’s Disease Regardless of Concomitant Small Vessel Disease
Source: J Alzheimers Dis. 2023 Jun 13;93(4):1537–49. doi: 10.3233/JAD-221187 (PMC10357154; doi:10.3233/JAD-221187)
Supplement: Supplementary Material [file jad-93-jad221187-s001.pdf]

Supplementary Material

Elecsys Cerebrospinal Fluid Immunoassays Accurately Detect Alzheimer's Disease Regardless of Concomitant Small Vessel Disease

Supplementary Table 1. Patient characteristics for the FDG-PET subgroup stratified by WMH

Characteristic	FDG-PET subgroup (*n*=72)	WMH low (<0.5 ml; *n*=28)	WMH medium (0.5–2.5 ml; *n*=24)	WMH high (>2.5 ml; *n*=20)
Mean age, y (SD)	63.7 (10.03)	58.0 (9.56)	66.0 (7.60)	68.8 (9.75)
Median age, y (IQR)	63 (55−72)	56 (50–64)	63.5 (60.25–72)	71.5 (61–75.75)
Male, *n* (%)	34 (47.2)	17 (60.7)	10 (41.7)	7 (35.0)
Mean CDR global (SD)	0.71 (0.32)	0.63 (0.22)	0.75 (0.36)	0.78 (0.38)
Median CDR global (IQR)	0.5 (0.5–1)	0.5 (0.5–0.875)	0.5 (0.5–1)	0.5 (0.5–1)
Mean CDR SOB (SD)	3.60 (2.48)	2.96 (1.30)	3.73 (2.56)	4.40 (3.47)
Median CDR SOB (IQR)	3.0 (2.0–4.5)	2.5 (2.5–3.0)	3.5 (1.50–5.0)	3.5 (2.5–4.5)
Mean WMH, ml (SD)	3.57 (8.60)	0.19 (0.15)	1.20 (0.52)	11.14 (13.85)
Median WMH, ml (IQR)	0.876 (0.216–2.730)	0.176 (0.062–0.328)	1.080 (0.828–1.434)	4.444 (2.880–17.960)
Mean $A\beta_{42}$, pg/ml (SD)	861.29 (402.79)	944.13 (451.07)	795.23 (369.02)	824.59 (367.76)
Median $A\beta_{42}$, pg/ml (IQR)	743.1 (560.275–1,069.5)	752.6 (589.85–1,427.5)	688.8 (521.425–898.675)	818.2 (528.325–1,040.5)
Mean $A\beta_{40}$, pg/ml (SD)	16,363.40 (4,922.18)	15,820.29 (50,10.31)	16,916.17 (5,212.58)	16,460.45 (4,597.92)
Median $A\beta_{40}$, pg/ml (IQR)	16,476 (12,768.25–19,177.75)	14,861.5 (12,241.25–18,961)	17,087.5 (13,460.75–20,839)	16,263.5 (13,164.25–20,683.5)
Mean pTau181, pg/ml (SD)	32.83 (19.31)	28.30 (16.68)	35.37 (18.33)	36.14 (23.28)
Median pTau181, pg/ml (IQR)	30.175 (18.51–37.79)	26.485 (15.23–35.5375)	31.66 (21.6225–42.58)	32.79 (19.335–40.2225)
Mean tTau, pg/ml (SD)	334.27 (184.65)	286.86 (146.91)	354.10 (166.20)	376.86 (239.98)
Median tTau, pg/ml (IQR)	313.05 (215.125–367.575)	278.00 (172.075–343.25)	320.45 (223.325–442.95)	352.55 (247.425–406.95)
Mean pTau181/$A\beta_{42}$ (SD)	0.05 (0.04)	0.04 (0.03)	0.05 (0.02)	0.06 (0.06)
Median pTau181/$A\beta_{42}$ (IQR)	0.04 (0.02–0.06)	0.04 (0.01–0.05)	0.05 (0.03–0.07)	0.04 (0.02–0.09)
Mean tTau/$A\beta_{42}$ (SD)	0.49 (0.38)	0.39 (0.30)	0.50 (0.22)	0.61 (0.58)
Median tTau/$A\beta_{42}$ (IQR)	0.41 (0.24–0.61)	0.38 (0.13–0.46)	0.51 (0.35–0.64)	0.36 (0.29–0.84)
Mean $A\beta_{42}/A\beta_{40}$ (SD)	0.05 (0.02)	0.06 (0.02)	0.05 (0.02)	0.05 (0.02)
Median $A\beta_{42}/A\beta_{40}$ (IQR)	0.05 (0.04–0.06)	0.05 (0.04–0.09)	0.04 (0.04–0.05)	0.05 (0.04–0.06)
FDG-PET positivity, *n* (%)	42 (58.3)	14 (50.0)	15 (62.5)	13 (65.0)

Aβ, amyloid-beta; AD, Alzheimer's disease; CDR, clinical dementia rating scale; CSF, cerebrospinal fluid; FDG-PET, fluorodeoxyglucose F18-positron emission tomography; IQR, interquartile range; *n*, number; pTau181, phospho-Tau181; SD, standard deviation; SOB, sum of boxes scores; tTau, total Tau; WMH, white matter hyperintensities; y, years.

Supplementary Table 2. Multivariate linear regression analyses between Elecsys CSF immunoassay biomarkers and WMH in patients with amyloid positivity

Dependent variable	corrR^2	Independent variables	Amyloid positive subgroup (n=43)[a]	
			beta	p
pTau181	-0.063	Aβ_{42}	-0.091	0.681
		WMH	0.012	0.979
		Aβ_{42} x WMH	0.063	0.888
pTau181	0.045	Aβ_{40}	0.326	0.094
		WMH	0.142	0.747
		Aβ_{40} x WMH	0.025	0.953
pTau181	0.375	Aβ_{42}/Aβ_{40}	-0.605	0.001*
		WMH	0.364	0.695
		Aβ_{42}/Aβ_{40} x WMH	-0.345	0.709
pTau181	0.749	tTau/Aβ_{42}	1.121	<0.001*
		WMH	0.132	0.520
		tTau/Aβ_{42} x WMH	-0.482	0.070
tTau	-0.057	Aβ_{42}	-0.032	0.882
		WMH	0.100	0.829
		Aβ_{42} x WMH	0.028	0.951
tTau	0.062	Aβ_{40}	0.333	0.084
		WMH	0.178	0.683
		Aβ_{40} x WMH	0.033	0.937
tTau	0.314	Aβ_{42}/Aβ_{40}	-0.530	0.003*
		WMH	0.579	0.553
		Aβ_{42}/Aβ_{40} x WMH	-0.512	0.598
tTau	0.970	pTau181	0.898	<0.001*
		WMH	0.532	0.292
		pTau181 x WMH	0.199	0.016*
		Age	0.082	0.021*
		Age x WMH	-0.819	0.067
		Sex	-0.002	0.945
		Sex x WMH	0.149	0.123
tTau	0.723	pTau181/Aβ_{42}	0.912	<0.001*
		WMH	-0.089	0.654
		pTau181/Aβ_{42} x WMH	-0.053	0.827

Aβ, amyloid-beta; CSF, cerebrospinal fluid; n, number; pTau181, phospho-Tau181; tTau, total Tau; WMH, white matter hyperintensities. [a]Patients with CSF Aβ_{42}/Aβ_{40} positivity. *Indicates statistical significance (p<0.05).

Supplementary Table 3. Univariate linear regression analyses between Elecsys CSF immunoassay biomarkers and WMH[a]

Dependent variable	Independent variable	Whole group (*n*=84)		
		beta	*p*	corrR^2
tTau	pTau181	0.978	<0.001	0.957
tTau	WMH	0.229	0.060	0.038
tTau	Age	-0.039	0.749	-0.014
tTau	CDR SOB	0.140	0.256	0.005
		Amyloid positive subgroup (*n*=43)[b]		
tTau	pTau181	0.977	<0.001	0.954
tTau	WMH	0.132	0.397	-0.006
tTau	Age	-0.236	0.128	0.032
tTau	Sex	0.096	0.539	-0.015

Aβ, amyloid-beta; CDR SOB, Clinical Dementia Rating scale sum of boxes scores; CSF, cerebrospinal fluid; *n*, number; pTau181, phospho-Tau181; tTau, total Tau; WMH, white matter hyperintensities. [a]Corresponding to the multivariate analyses (Table 6 and Table S2), in which WMH were a significant predictor or modifier of the associations between CSF biomarkers. [b]Patients with CSF $A\beta_{42}/A\beta_{40}$ positivity.
